# A lower-limb motor imagery BCI using virtual reality and novel calibration strategy in post-stroke patients

**DOI:** 10.1007/s11517-025-03497-6

**Published:** 2026-02-11

**Authors:** Leticia Silva, Jéssica Lima, Denis Delisle-Rodriguez, Teodiano Bastos-Filho

**Affiliations:** 1 Postgraduate Program in Electrical Engineering, Universidade Federal do Espírito Santo, Av. Fernando Ferrari, 514, Vitória, 29075-910 Espírito Santo Brazil; 2 Postgraduate Program in Biotechnology, Universidade Federal do Espírito Santo, Av. Marechal Campos, 1468, Vitória, 29047-105 Espírito Santo Brazil; 3 Postgraduate Program in Neuroengineering, Edmond and Lily Safra International Institute of Neurosciences, Av. Alberto Santos Dumont, 1560, Macaíba, 59288-899 Rio Grande do Norte Brazil

**Keywords:** Brain-computer interface, Lower-limb rehabilitation, Motor imagery, Riemannian geometry, Stroke

## Abstract

**Abstract:**

This study proposes a novel two-step calibration strategy in a Motor Imagery (MI)-based Brain-Computer Interface (BCI) system for lower-limb post-stroke patients rehabilitation, using electroencephalography (EEG) signals, a virtual reality serious game, and a pedal end-effector. This research proposes a novel MI feature extraction algorithm combining EEG data from pedaling MI and actual movements using k-Nearest Neighbors (k-NN) and probability analysis. The extracted features are used to re-calibrate the BCI system, and improve its accuracy. Initially, participants performed 20 MI trials without feedback (open-loop, Calibration Mode #1) while receiving passive movement. During this phase, EEG data were processed using the Riemannian Geometry method to train a Linear Discriminant Analysis (LDA) classifier. In the second step (closed-loop, Calibration Mode #2), feedback through passive movement was provided, changing the pedal’s speed according to the MI classification consistency. A chronic post-stroke patient tested the proposed BCI system, first receiving transcranial Direct Current Stimulation (tDCS) before each session. The calibration strategy improved classification accuracy from 52.60% (Mode #1) to 86.74% (Mode #2). The classification remained effective throughout sessions, allowing for immediate feedback-driven training while collecting more reliable EEG MI data. The BCI developed here is able to provide post-stroke patient rehabilitation by engaging lower-limb central and peripheral mechanisms through simultaneous MI and passive pedaling during extended workout sessions.

**Graphical abstract:**

**Figure:**
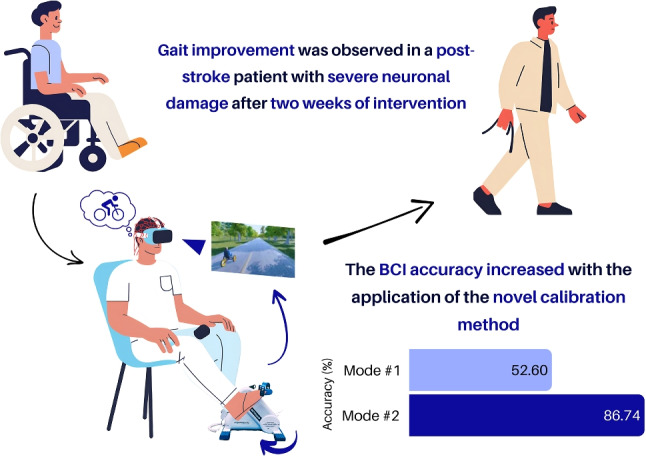
**Novel BCI Calibration Enhances Post-Stroke Gait Rehabilitation**. Implementation of a high-accuracy calibration method (86.74%) in a closed-loop BCI system facilitated rapid gait recovery in a patient with severe neuronal damage following a two-week intervention protocol.

## Introduction

Stroke is a leading cause of death and disability worldwide, often resulting in unilateral motor impairments that compromise gait [1, 2]. Although traditional therapies, such as mirror therapy, may enhance motor functions, they do not fully restore normal gait [1–3]. Thus, emerging technologies such as Brain-Computer Interfaces (BCIs) are of growing interest for assisting or rehabilitating patients unable to voluntarily initiate movement [1]. Non-invasive BCIs create communication pathways for individuals with severe motor disabilities by acquiring and translating brain activity into commands for robotic assistance and/or sensory feedback [4]. Electroencephalography (EEG) is the most common technique due to its low cost, portability, and high temporal resolution. Consequently, numerous studies have explored EEG-based Motor Imagery (MI) for stroke rehabilitation, proposing diverse preprocessing, feature extraction, and classification methods [1, 2, 5–7].

Motor Imagery (MI) can be classified as Kinesthetic (KMI), based on imagined haptic sensations, or Visual (VMI), based on visualizing movements [8]. Both forms can stimulate neural networks via mirror neuron activation and support motor recovery through MI-based BCIs [6, 9, 10]. However, patients with severe impairments often have difficulty performing KMI due to cognitive or motor deficits [11]. Virtual Reality (VR) has been proposed to overcome these limitations by immersing patients in engaging simulated environments, facilitating MI execution for upper and lower limbs and activating visuomotor processes related to action, observation, and simulation [9, 11–13]. For example, Vourvopoulos et al. [2] presented a MI-based BCI combined with VR for post-stroke upper-limb rehabilitation, showing improvements in motor function.

Our research group previously built a low-cost EEG-based BCI system [1], which allows post-stroke patients to command a customized motorized pedal through pedaling MI and receive passive movement feedback. Unlike expensive robotic systems such as exoskeletons, pedaling exercise systems are affordable alternatives that show promising results for lower-limb motor recovery and gait [14, 15]. However, like other MI-based BCIs, our earlier approach requires long calibration times, limiting clinical use. In addition, EEG motor rhythms have low amplitudes and poor Signal-to-Noise Ratio (SNR) [1, 16], while MI-related patterns may vary with the individual’s neural condition. Consequently, unreliable outputs from inaccurate classification models may be unconsciously applied, leading to unsuccessful neural interventions. In contrast, studies show that BCIs with good accuracy and low computational cost enhance closed-loop interaction between user and device, promoting neuroplasticity [1, 17].

Thus, our current study introduces an MI-based BCI with reliable feedback and significant reduced calibration-time consumption to provide a real closed-loop, promote neuroplasticity, and consequently facilitate its clinical use. Moreover, this research also introduces a system with a calibration strategy that stimulates central and peripheral mechanisms associated with lower limbs end-to-end, using transcranial Direct Current Stimulation (tDCS), pedaling MI, and passive pedaling movement. To extract reliable features, a two-step feature extraction method on the Riemannian Geometry (RG) space is used, which includes *k*-Nearest Neighbors (k-NN) with a probability analysis for obtaining features over EEG periods in which the individual probably performed MI tasks. In addition, Linear Discriminant Analysis (LDA) is used for MI recognition. We are motivated to propose a solution that addresses current scientific issues within the experimental protocol, as our aim is to enhance data reliability and reduce calibration time, while also increasing patient involvement. Our research contributes by identifying MI patterns that closely resemble real movements, leading to improved feature extraction, analysis, and a more accurate classifier model. This enhances the effectiveness of the BCI intervention and may prove attractive for chronic post-stroke rehabilitation, as it also helps reduce patient boredom. This work represents an initial, exploratory investigation into the feasibility and potential of this novel BCI system.

The next sections of this paper are structured as follows. Section 2 describes first the proposed BCI system to provide mental and physical pedaling exercises. Section 3 presents the experimental protocol and the methodology used for evaluation. Afterward, the results and discussions are presented in Sections 4 and 5, respectively, comparing the findings with the state-of-the-art. Finally, the conclusion is given in Section 6.

## Proposed brain-computer interface

**Fig. 1:**
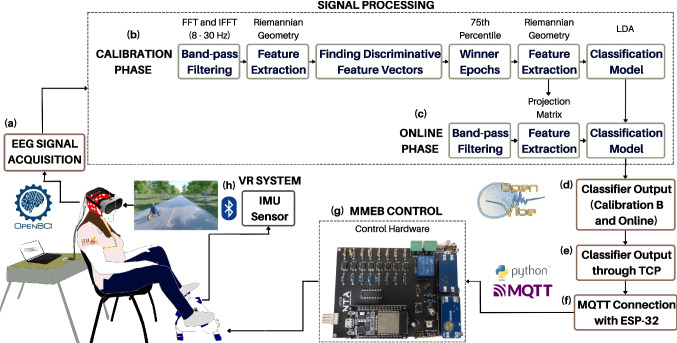
MI-based BCI system for EEG processing and pedaling MI recognition, providing real and virtual movements

Figure 1 illustrates the proposed MI-based BCI and VR with pedal end-effector, which is divided into four main sub-systems: 1) EEG signal acquisition; 2) signal processing and MI classification; 3) Customized Mini Motorized Exercise Bike (MMEB) with communication and control interface board; and 4) VR environment. This system using OpenViBE acquires and processes EEG signals, and also recognizes pedaling MI. The classification’s outputs are sent via a communication and control interface board by which the individual commands the MMEB speed. The MMEB pedal movement captured through an attached in-house Inertial Motion Unit (IMU) sensor with bluetooth connection, synchronizes with an avatar pedaling in a VR-based Serious Game (SG) software developed in our research group. It runs in another computer to offer immersive visual feedback to the individual. For this purpose, both a script developed in Python 3 and an MQTT protocol are employed. It is worth noting that OpenViBE, Python 3 and the MQTT broker run simultaneously on a single laptop with the following hardware features: Intel® Core ™ i77700HQ (2.8 GHz to 3.8GHz 6 MB L3 Cache) Intel HM175, RAM 16 GB DDR4 2400 MHz, NVIDIA® GeForce® GTX1060 with 6 GB GDDR5 memory.

As illustrated in Fig. 1, this MI-based BCI system proposed here consists of different phases: a calibration phase and one online phase. The former processes the EEG signals collected in the rest state, MI and passive pedaling to create a classification model for MI recognition. The calibrated system is later used in the online phase for the individual to command the customized MMEB device and receive pedaling movements. As a novelty, it is proposed here a calibration strategy that can be applied through two operation modes, as explained in the next subsection. Furthermore, the other subsections provide a detailed explanation about the BCI stages and modules.

**Fig. 2:**
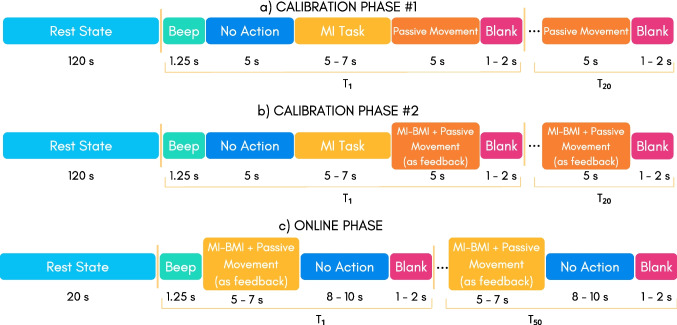
MI-based BCI scheme. (**a**) Calibration phase #1; (**b**) Calibration phase #2; (**c**) Online phase

### Operation modes

#### Mode $\#$1

As aforementioned, our BCI operates by using a calibration composed of two phases and finally one online phase. In the first calibration stage (Phase $\#$1), a period of 2 min with the individual remaining in the rest state is initially recorded, immediately followed by a sequence of 20 trials, each one separated by a blank space of 1$\sim$2 s, as shown in Fig. 2(a). Each trial is composed of a period of a 1.25s-beep sound (trial start), and other three periods in which the individual first rests for a period of 5 s, after performs pedaling MI in open-loop (no feedback) for a total of 5$\sim$7 s, and finally undergoes passive movements completing 5 s.

In the second calibration stage (Phase $\#$2), the individual completes a period of 2 min remaining in the rest state, and a sequence of 20 trials now using the BCI calibrated with EEG data from Phase $\#$1, as shown in Fig. 2(b). Thus, the individual now performs pedaling MI in a closed-loop (with feedback) by commanding the MMEB’s speed.

Finally, the Online phase is composed of a period of 2 min in which the individual remains resting, and after performs a total of 50 trials employing the BCI re-calibrated with all data from Phase $\#$1 and Phase $\#$2. Each trial first provides a 1.25s-beep sound (trial start), indicating for the individual to execute pedaling MI in a closed-loop for a total of 5$\sim$7 s, followed by an 8$\sim$10s-rest sate, and a 1$\sim$2s-blank space, as shown in Fig. 2(c). Here, the individual commands the MMEB’s speed between 30 and 60 rpm, which gradually increases towards the maximum value while the BCI correctly classifies the participant’s MI, or decreases backwards the minimum speed otherwise.

It is worth mentioning that the projection matrix and classifier’s model are saved in this last stage, which can be used again another day, such as explained in the next subsection.

#### Mode $\#$2

Here, the BCI system directly begins using the Phase $\#2$ (see Fig. 2(b)) with the projection matrix and classification model saved on the previous day. Afterwards, the system is newly calibrated only with the current collected EEG data, computing a new projection matrix and classification model to conduct again the Phase $\#2$. After finishing this stage, the BCI is re-calibrated using the full dataset (only EEG data acquired on the current day) from both phases, and the Online phase is later carried out (see Fig. 2(c)). As advantages, this other operation mode begins the intervention, collecting more reliable EEG data for calibration while the participant executes MI and simultaneously receives feedback. Moreover, the participant starts the intervention activating end-to-end central and peripheral mechanisms associated with lower-limbs. It may engage better the participant throughout the intervention, increasing his/her motivation.

### Signal acquisition

The open-source OpenBCI Cyton and Daisy Board are used here to acquire 16 EEG channels with a sampling rate at 125 Hz using the OpenVibe software, also compatible with Python [1], as shown in Fig. 1(a). This wireless acquisition system has a 32-bit processor, which communicates with a computer via an OpenBCI USB dongle by using the RFduino radio module. The EEG signals are acquired through a cap of 64 wet Ag-AgCl electrodes, but only electrodes over FP1, FP2, F3, F4, FC1, FC2, C3, Cz, C4, CP1, CP2, P3, Pz, P4, P7 and P8 according to the 10-10 international system, are used in this study, as done in previous research [1, 5–7, 18]. The reference (A1) and ground (A2) are placed on the left and right earlobes, respectively.

### Pre-processing

EEG epochs of 1s each 64 ms are band-pass filtered in a frequency range from 8 to 30 Hz during both the Calibration and On-line phases, as shown in Fig. 1(b)-(c), to remove unwanted noise and artifacts (such as electrooculogram), and also preserve the components of interest [1, 7, 8, 13, 18]. The filter applies the Fast Fourier Transform (FFT) and the inverse FFT [1] to first convert the EEG signals into the frequency domain, gives zero values for frequency components outside the desired range by using a rectangular function, and finally recovers the filtered signals into the time domain [1].

### Feature extraction

People with severe neural impairments, and consequently motor disability, can have enormous difficulty to execute MI accurately. Therefore, EEG data related to MI from these people may have intrinsic high uncertainty, as many periods in the signals may not carry information linked to MI tasks. To tackle this issue, we apply here our proposed calibration strategy [18] under the scheme shown in Fig. 2(a)-(b) to better extract features over periods of time in which individuals probably performed well MI without feedback. Our approach is composed of the following stages:

1. Calibration #1: An MI EEG record, termed RECORD1, also including data from rest state and passive movement is used here to calibrate the proposed BCI. This step is carried out by using both **E** and **E**$^r$ vectors, where **E** is composed of filtered EEG epochs of 1 s from the first 80% of all rest state data and KMI without feedback, whereas **E**$^r$ contains filtered EEG epochs of 1 s from the last 20% of all rest state data and passive movements. In short, **E**$^r$ is used as reference to find a subset $\tilde{{\textbf {E}}}$ $\subset$ **E** in which KMI features are more spatially associated with those features extracted in periods of passive movements. Then, this subset is used to obtain the best project matrix $\tilde{{\textbf {P}}}_{ref}$, feature vectors, and classification model.
2. Calibration #2: Another MI EEG record, termed RECORD2, now including data from rest state and MI with passive movement as feedback, is employed here to re-calibrate the proposed BCI. For this purpose, both RECORD1 and RECORD2 are concatenated to re-calibrate the BCI by repeating again the same steps implemented in Calibration #1. It is worth mentioning that the periods of 5 s containing EEG data from Passive Movements and MI + Passive Movements (as feedback) from both records are annotated with the same label. Then, **E** is now composed of filtered EEG epochs of 1 s from the first 80% of all rest state data and KMI without feedback, whereas **E**$^r$ now contains filtered EEG epochs of 1 s from the last 20% of all rest state data, passive movement, and MI+passive movement as feedback. As a result, the updated projection matrix and classification model consider EEG patterns obtained in both calibration phases.

In [18], the proof-of-concept of this approach for feature extraction reached the best performance on three existing MI databases by using the Riemannian Covariance Matrices (RCM) [1, 18–20]. Then, RCM is applied here by using the functions *covariances*, *meancovariances*, *Tangentspace* available at.^1^ More details in a pseudo-code about our implementation using RCM can be reviewed in [18].

Firstly, the Calibration #1 is explained in details as follows. Notice that a similar procedure is carried out in the Calibration #2. Let **C**={**C**$_1$,...,**C**$_i$,...,**C**$_N$} $\in \mathbb {R}^{N \times m \times m}$ be a set of covariance matrices from **E** each instant of time *i* ($i=1,2,\dots ,N$). From this set using a *k*-folds cross-trials validation ($k = 10$), the projection matrix ${\textbf {P}}_{ref} \subset$ **P** $\in \mathbb {R}^{m \times m}$ onto the tangential space that provides more discriminant MI features, and therefore the highest kappa value by using LDA is obtained [18]. **P**={**P**$_1$,...,**P**$_k$,...,**P**$_{10}$} is a set of projection matrices calculated in the cross-validation, where *k* takes values from 1 to 10 [18]. Although the ${\textbf {P}}_{ref}$ selection is guided by the highest kappa value, notice that the use of 10-fold cross-trials validation ensures tests across random multiple partitions of the data, thus, mitigating overfitting and supporting the model generalization to unseen EEG data.

In sequence, ${\textbf {P}}_{ref}$ is used on the full training set composed of **E** and **E**$^r$ to project their corresponding covariance matrices onto the Riemannian space, and obtain the feature training set. Let **X**={**x**$_1$,...,**x**$_i$,...,**x**$_N$} $\in \mathbb {R}^{N \times f}$ be a feature set, calculated after projecting the covariance matrices from **E** each instant of time *i* ($i=1,2,\dots ,N$). **x**$_i$ contains *f* features and a assigned class $y_i \in$ {1,2}, where this latter can be the rest state or pedaling KMI without feedback. Furthermore, let **X**$^r$ = {**x**$^r_1$,...,**x**$^r_j$,...,**x**$^r_M$} $\in$ $\mathbb {R}^{M \times f}$ be other feature set from **E**$^r$ each instant *j* ($j=1,2,\dots ,M$). **x**$^r_j$ also contains *f* features and an assigned class $y^r_j \in$ {1,2} that can be real pedaling passive movement without KMI or rest state.

The probability of a feature vector from KMI without feedback to come from the rest state can be computed by employing (1)-(3).

1 $$\begin{aligned} d_{ij}= & \sqrt{({\textbf {x}}_{i}-{\textbf {x}}^r_j)({\textbf {x}}_{i}-{\textbf {x}}^r_j)^T},\end{aligned}$$

2 $$\begin{aligned} p_{ik}= & \frac{\exp (-d_{ik})}{\displaystyle \sum _{k}\exp (-d_{ik})},\end{aligned}$$

3 $$\begin{aligned} p_{i}= & {\displaystyle \sum _{k} p_{ik} \times c_{ik}}~~,~~c_{ik}=1~\text {for }~y_{i}\ne y^r_{k};~0~\text {others} \end{aligned}$$

where *i* takes values from 1 to *N*, *j* spans from 1 to *M* for each *i*, and $d_{ij}$ is the Euclidean distance between **x**$_i$ and **x**$^r_j$. ${\textbf {x}}^r_k \subset {\textbf {x}}^r$ is the nearest neighbors ($k=1,2,\dots ,15$) around **x**$_i$, and $p_{ik}$ is then the probability of **x**$_i$ and its *k*th nearest neighbors to correspond to a same class. $p_i$ is the probability of **x**$_{i}$ be a pattern coming from other class.

As other step, the median of $p_i$ values corresponding to the same class *l* is used as a threshold $T_{l}$, to after selecting those *i* EEG epochs from **E** with high probability (with $p_i \le T_{l}$) of corresponding to the class *l*. This subset $\tilde{{\textbf {E}}}$ of selected EEG epochs from all classes *l*={1,2} is then used to compute its set of covariance matrices $\tilde{{\textbf {C}}}$={$\tilde{{\textbf {C}}_1}$,$\tilde{{\textbf {C}}_2}$,...,$\tilde{{\textbf {C}}}_Q$} $\in \mathbb {R}^{Q \times m \times m}$, and update the projection matrix $\tilde{{\textbf {P}}}_{ref}$. Finally, $\tilde{{\textbf {P}}}_{ref}$ is used on $\tilde{{\textbf {C}}}$ to obtain a new feature set $\tilde{{\textbf {X}}}$ $\in \mathbb {R}^{Q \times f}$. Notice that $\tilde{{\textbf {P}}}_{ref}$ is also used for feature extraction over covariance matrices of 1s-EEG epochs from the testing set (Evaluation phase), and during the Online phase.

### Classification

Classifiers with linear discriminant models are widely used in MI-based BCI systems that use EEG signals [1, 18, 21]. These methods use linear functions to distinguish different MI tasks from EEG patterns or features, allowing then individual’s MI classification in BCIs, translating it into control commands for assistive devices. In our study, we employ the LDA method, which is a computationally efficient classifier, widely used with success in BCI systems for MI recognition. This method utilizes hyperplanes to differentiate between data representing different classes [1, 21]. Such as aforementioned in Section 2.4, this classifier is used to obtain both ${\textbf {P}}_{ref}$ and $\tilde{{\textbf {P}}}_{ref}$. Furthermore, LDA is used here on the new feature set $\tilde{{\textbf {X}}}$ to train a model, which is after extended to classify unseen data.

### Motorized pedal and computer interface

A commercial motorized pedal (Exerpeutic ACTIVcycle) was customized with in-house hardware featuring an ESP32 microcontroller to receive brain commands wirelessly for lower-limb rehabilitation. The ESP32 microcontroller communicates wirelessly with a laptop running OpenViBE software, enabling a control of pedal speed (30–70 rpm). The hardware components include optocouplers, which ensure high isolation and protection against interference or short circuits. Compared to our previous study utilizing a Raspberry Pi 3B [1], the ESP32-based hardware offers a low-cost solution with wireless communication to a laptop, where raw EEG signal acquisition and processing are conducted to identify the individual’s MI.

### Communication and control

Such as aforementioned, an ESP32 is used to control the MMEB’s speed according to the output from the MI classification system running in OpenViBE. Communication between OpenViBE and ESP32 is established using Python3 and the MQTT protocol, enabling: 1) a beep sound to guide individuals during Calibration and Online phases; and 2) translation of classification outputs (rest or pedaling MI) into speed control commands. The classifier’s output is sent via TCP to a Python3 script, which adjusts the MMEB’s speed. Starting at 30 rpm, the speed increases if the array sum $\sum _{i=1}^n v_i \ge \frac{n}{2}+1$ (pedaling MI outputs) or decreases otherwise, with a maximum speed configurable by a physiotherapist ranging from 30 to 70 rpm based on patient needs.

### Virtual reality

A SG designed in [22] for lower-limb rehabilitation is used here, leveraging the VR immersive properties [22]. Both VR and MMEB are connected and synchronized via a wireless IMU sensor with bluetooth connection. The VR-SG is simultaneously displayed to the individual in a pair of HTC VIVE glasses and a computer monitor, providing visual feedback to the individual while he/she performs MI, controls the MMBE’s speed, and observes the reached distance and speed. Thus, the integration of VR in the SG enhances the individual’s experience and provides a more dynamic and engaging approach for lower-limb rehabilitation.

## Experimental protocol

### Post-stroke patient

This study was approved by the UFES/Brazil Research Ethics Committee (CAAE: 46099421.9.0000.5542). The experimental protocol was carried out at the Physical Rehabilitation Center of Espirito Santo (CREFES), and the post-stroke patient was selected by following the inclusion and exclusion criteria as follows.

As inclusion criteria of this research, patients were considered in the subacute phase to chronic post-stroke, between 1–12 months after the injury, aged over 18 years old. As other inclusion criteria, they should have a stable general medical condition, and ability to understand and follow instructions that are assessed by the Mini-Mental State Examination. Additionally, the patients should be also able to stand with or without support.

Patients are not incorporated in this research because of the following exclusion criteria: scored above 2 on the Ashworth Scale, had autonomic dysreflexia, pressure ulcers, thrombosis, peripheral neuropathy, severe aphasia, severe perceptual problems or other neurological conditions, had lower-limb surgeries in the last 6 months, had severe osteoporosis, skin lesions at the electrode site, very limited visual ability, more than one stroke episode, decompensation in psychiatric disease, decompressive craniectomy, or if they did not sign the Informed Consent to voluntarily participate of this research.

The selected post-stroke patient (PS1) (male, 73 years old, with 2 months of ischemic stroke, and right hemiparesis) and family were firstly informed about the research, to after voluntarily read and provide signed the Informed Consent Form to participate in the study.

### Procedure

The protocol consists of four stages: 1) familiarization with the experiment (5 min); 2) tDCS preparation and application for a total of 20 min, with a current intensity of 2 mA; 3) EEG electrode preparation with duration of 15 min; 4) VR glasses configuration and calibration with the SG (5 min); and 5) protocol execution (approximately 35 min).

tDCS has shown to be a promising approach in neuromuscular rehabilitation of individuals who have suffered a stroke. tDCS involves the application of low-intensity electrical current to the cerebral cortex, with the aim of modulating cortical excitability and facilitating neuronal plasticity. Recent clinical studies have demonstrated that tDCS can improve motor function and muscle strength in post-stroke patients, in addition to promoting beneficial neuroplastic changes in the motor cortex [6, 23]. These findings underscore the importance of tDCS as a non-invasive and potentially effective intervention, offering another perspective for optimizing the functional outcomes of patients in this research.

In our experiments, the patient was invited to seat in a comfortable chair with their feet on the floor, wearing first a cap with two electrodes (anode and cathode) following the dual-tDCS mode. The anode and cathode are placed on M1 of the affected hemisphere, and the cathode positioned on the cerebellum of the unaffected hemisphere. The application of tDCS was randomized to ensure a controlled experimental setup. Afterwards, the patient weared a 64-EEG cap with 16 electrodes and a pair of VR glasses, also positioning their feet on both MMEB pedals. During the EEG preparation, the patient’s scalp skin was first prepared with an abrasive gel before attaching electrodes, and after employing a gel to reduce the skin-electrode impedance, keeping it below 10 k$\Omega$. We utilized here the OpenBCI Graphical User Interface (GUI) to check the impedance.

As a first stage, the patient undergo a familiarization step with the protocol, where he was instructed to practice cycling MI on a beach or in an indoor environment, such as a gym. To save time, the VR glasses were then simultaneously placed on the patient’s head, and a calibration of both the VR environment and the SG was conducted. Subsequently, the experiment was conducted using the proposed MI-based BCI, while the brainwave data, linked to a pedaling KMI task, were collected. Operation Mode #1 was employed during the initial day of the experimental week followed by Operation Mode #2 for the subsequent days of the week. Breaks of 5 min between the calibration phases and online phase were given along the experiment. The complete experimental setup, including the familiarization and all protocol execution, had a duration of 1 h and 35 min.

### Data description

The EEG recordings from the post-stroke patient are composed of three classes: rest state, pedaling MI, and passive pedaling. The patient recruited in this study completed two calibration runs, each one with a total of 20 trials. The EEG data from both calibration phases were then concatenated and split into a training set and a testing set by using a 10-fold cross-trial validation. For calibration, EEG epochs of 1 s were extracted from the raw EEG, where the periods related to the rest state were obtained from the first 2 min of each phase (see Fig. 2a-b), whereas the data linked to pedaling MI and passive movements were extracted in each trial from the last 3 s and the first 3 s of their corresponding periods (see Fig. 2a-b), respectively.

**Fig. 3:**
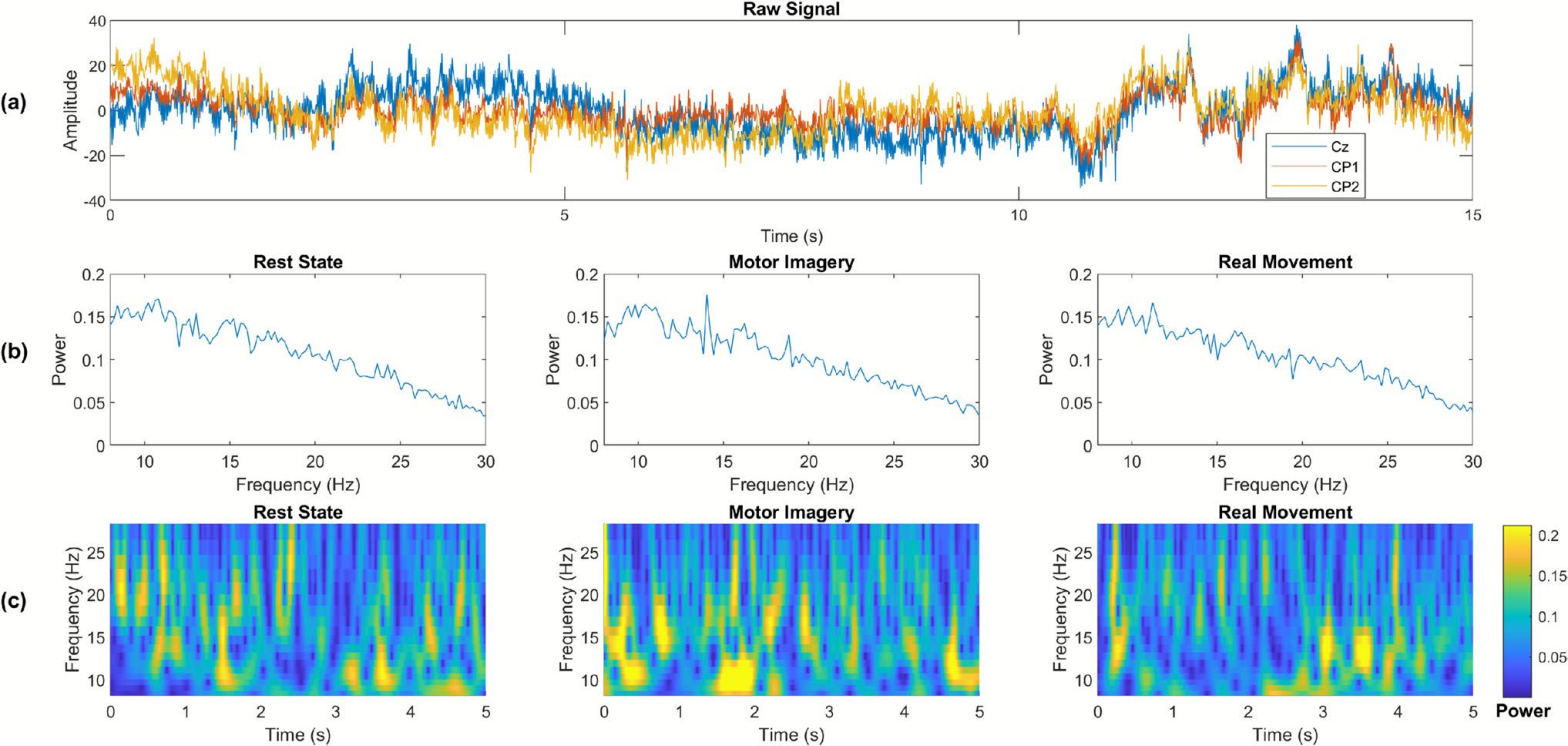
One-trial EEG calibration signal. (**a**) Raw signal over Cz, CP1 and CP2; (**b**) Power and frequency representation over Cz for rest state, MI and real movement; (**c**) Time-frequency analysis over Cz for rest state, MI and real movement

## Results

### EEG signal assessment and analysis

Figure 3(a) shows the EEG signal captured during a single trial over Cz, CP1, and CP2 locations. The recordings have a duration of 15 s, with the initial 5 s related to rest state, followed by 5 s of MI, and concluding with 5 s of real movement. It is noteworthy that the developed hardware allowed for reducing noise in the raw signal, consequently enhancing the SNR and improving the overall quality of the EEG recordings. To further analyzing the signals in the time-frequency domain, each trial over Cz underwent the Common Average Reference (CAR) spatial filtering to reduce common noise sources (see Fig. 3(b)). Then, the Continuous Wavelet Transform (CWT) based on Morlet (Gabor) wavelet was employed to extract the signal’s time-frequency information. Finally, the average time-frequency representation was computed over all trials. Figure 3(c) illustrates the result for rest state, MI, and real movement. Notice that a significant increase in power can be observed during the MI task in mu (8–12 Hz) and low beta (13–22 Hz) bands.

**Fig. 4:**
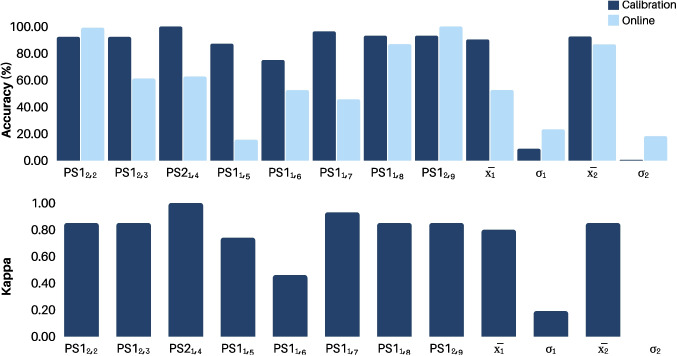
Performance achieved for both Calibration and Online phases in terms of accuracy and kappa index

### Calibration phase analysis

Both accuracy (ACC) and kappa ($\kappa$) metrics were used here to evaluate the performance of our BCI system, such as done by other studies [1, 5, 24]. ACC measures the ratio of correctly classified instances in the testing set with respect to all data classes, summarizing the overall performance. On the other hand, $\kappa$ is used to assess the agreement between the predictions and their corresponding true labels. Figure 4 shows the performance of our MI-based BCI system in classifying between MI and rest state, which utilizes discriminant pattern recognition and two calibration operation modes. The extracted features are then applied to a LDA classifier for pedaling MI recognition. A post-stroke patient (PS1) with hemiparesis tested the BCI approach. A notation was employed to indicate the use of either Mode $\#$1 or Mode $\#$2, identified by the day of use. For instance, PS1$_{1,3}$ represents the use of Mode $\#$1 on the third day for the patient PS1, whereas PS1$_{2,1}$ denotes the use of Mode $\#$2 on the first day for Patient PS1. In addition, $\bar{x}_1$ and $\sigma _1$ denote the average and standard deviation, respectively, using Mode $\#$1.

It is worth mentioning that EEG data were collected from Patient PS1 for nine days. Training procedures for Mode #1 were executed without issue for Patient PS1, ensuring enough data. However, some technical issues during the Online phase hindered its execution. Despite these issues, training data was successfully collected, and the classifier was trained for Mode #1. Consequently, the classifier’s model derived from this training was employed on the subsequent day within the context of Mode #2. Then, the results of the online phase are not presented in Fig. 4.

The second calibration method (Mode $\#$2) was applied on three different days for Patient PS1. PS1$_{2,2}$ and PS1$_{2,3}$ achieved a similar performance, with an ACC of 92.2% and $\kappa$ of 0.85. Furthermore, a similar ACC value was observed for PS1$_{2,9}$ (ACC = 93.18% and $\kappa$ = 0.85). To compare both calibration schemes, the average and standard deviation were computed for each scheme, as shown in Fig. 4. A similar performance, in terms of ACC, was observed in both Mode $\#$1 and Mode $\#$2, with $\bar{x}_1$ = 90.35% ($\sigma _1$ = 8.75%) and $\bar{x}_2$ = 92.55% ($\sigma _2$ = 0.44%), respectively.

### Online phase analysis

Such as aforementioned, the classification model derived from the BCI Calibration phase was employed during the Online phase to classify when the patient was performing pedaling MI, controlling the MMEB speed in a closed-loop neural system. An individual model was generated for each patient. It is worth noting that 1s-epochs were processed sequentially with a 0.065-s overlap over a period of 5$\sim$7 s after the beep sound. Notice also that the MMEB device is triggered at the same time, as the pedaling MI (following the beep), and the speed can be modified as more MI patterns are accurately classified.

Figure 4 shows the online performance of the EEG-based MI-BCI system. Regarding Patient PS1, ACC of 86.77% was achieved on the eighth day (PS1$_{1,2}$) by using Mode $\#$1. However, PS1$_{1,5}$ was not successful, with a very low ACC of 15.51%. On this day, the patient had difficulty understanding the tasks (resting and MI) during the experiment, which affected the results. In contrast, Mode $\#$2 demonstrates a good approach, as shown in Fig. 3. ACC up to 100.00% was observed for PS1$_{2,9}$, as well as 99.11% for PS1$_{2,2}$. Likewise the Calibration phase, we computed the average and standard deviation for Online phase. In Fig. 4, it is worth noting that Mode $\#$2 (ACC = 86.74%) outperformed the Mode $\#$1 (ACC = 52.60%). This result suggests that our novel calibration method may in fact improve the MI-based BCI performance.

## Discussion

The use of BCIs in motor rehabilitation has shown promising results, particularly for individuals with lower-limb impairments who can employ KMI to control external devices. In clinical settings, BCI training has demonstrated effects comparable or superior to commonly used interventions, such as mirror therapy, since it establishes a closed-loop pathway where brain signals directly interact with external devices [25]. In addition, studies on upper limbs suggest that VR can significantly improve motor recovery in post-stroke patients, providing strong preliminary evidence of its focused effectiveness [26].

Several studies using systems or BCIs based on pedaling movements for lower-limbs rehabilitation have been proposed [1, 18, 27, 28]. As an example, the use of passive movements for calibration in a KMI classification system was investigated in [27]. That study employed an upper-limb BCI system with 27 EEG channels to evaluate both healthy subjects and post-stroke patients during passive movements without feedback through a haptic knob robot. The authors proposed an adaptive strategy to recognize MI tasks, reporting a mean ACC of 69.78% on healthy subjects by using this method, compared to 65.13% without it. Similarly, the mean ACC for post-stroke patients increased, achieving ACC values of 78.70% and 67.10%, respectively. Corroborating our hypothesis, a previous study conducted by [28], investigated the use of passive and active movements from left and right hands to train a Support Vector Machine (SVM) classifier. The trained classifier was subsequently utilized to recognize left and right hand MI of 15 healthy subjects. The study reported mean ACC of 68.7% (using passive movement), 69.56% (using active movement), and 71.61% (using MI). These authors concluded that the KMI network characteristics were similar to those observed during passive movements. In addition, recent clinical BCI studies confirmed reliable classification performance in lower-limb tasks, with [29] and [30] reporting consistent ACC values during training sessions with stroke patients.

The main objective of our study is to improve BCI calibration by considering criteria beyond classification performance, aiming for a system more suitable for clinical practice and addressing limitations of existing approaches. One major drawback of current BCIs is the long protocol duration, with an average of about 5h [31], which may reduce patient motivation and impair performance. In our previous study [1], for example, low performance may have been linked to reduced motivation during a 2h experiment. In contrast, our novel BCI setup lasts 1h 35min, representing a 21% reduction compared to the earlier protocol.

On the other hand, other approaches provide a closed-loop calibration step only using visual feedback [18]. Here, we presented a BCI with calibration step that include passive movement and MI in both Modes #1 and #2, but the last mode provides a closed-loop with physical exercise and visual feedback in a virtual environment. Also, the Mode #2 uses the classification model from a previous day to immediately promote the stimulation of central and peripheral mechanisms while EEG data are collected for a new calibration and obtain a more robust model.

In our previous work [18], we evaluated the same MI-based BCI calibration approach to enhance classification outputs more related to individual’s motor intention. To conduct the evaluation, we employed an in-house dataset involving eight healthy volunteers and two post-stroke patients, all of whom participated in lower-limb MI tasks and subsequently received passive movements as feedback. Furthermore, we utilized well-established public EEG datasets, including the BCI Competition IV dataset IIb, among others, featuring upper and lower-limb motor imagery tasks performed by healthy subjects with continuous visual sensory feedback. The system proposed, based on the two-step Riemannian Geometry method, significantly outperformed Baseline methods, achieving an average accuracy of up to 82.29%.

Although this study focuses on our proposed MI-based BCI, clinical outcomes of a post-stroke patient using the same system are detailed in our recent work [32]. This patient showed significant power changes in Mu (8–12 Hz), low beta (13–22 Hz), and high beta (23–35 Hz) rhythms, a 39.99% reduction in steps per minute during a 10-meter walking test, and improvements in lower-limb physical and motor functions according to the Fugl-Meyer Assessment. Our BCI approach in Mode #2 employed the classification model generated from the previous day, optimizing calibration and promoting neuromotor activation in central and peripheral mechanisms. A practical limitation, however, is that if patients do not properly understand or perform MI during calibration, the discriminative quality of the data may decrease, potentially affecting later phases. This effect may be partially mitigated by confidence-based epoch selection and passive-movement feedback. Moreover, as this is a single-subject preliminary study, the generalizability of the findings is limited, reinforcing the need for future research with larger and more diverse cohorts.

## Conclusion

This preliminary study presents an MI-based BCI system integrating VR, serious games, tDCS, and a pedal end-effector for post-stroke rehabilitation. A novel two-stage calibration strategy was developed to enhance patient engagement during pedaling MI tasks. Validation with a post-stroke patient demonstrated the system’s capability to establish a real closed-loop, allowing the patient to control the motorized pedal. These preliminary results suggest that this BCI system has the potential to advance neurorehabilitation strategies and support motor recovery in clinical applications.

## Data Availability

The material analyzed during the current study is not publicly available due to its content of sensitive personal data. Datasets generated may be available on request, after ethical considerations.

## Footnotes

1 https://github.com/alexandrebarachant
